# Canadian Youth Preferences for E-Cigarettes: A Discrete Choice Experiment

**DOI:** 10.1177/1179173X251322597

**Published:** 2025-03-11

**Authors:** Daniel Eisenkraft Klein, Jiamin Shi, Robert Schwartz

**Affiliations:** Dalla Lana School of Public Health, 274071 University of Toronto, Toronto, ON, Canada

**Keywords:** discrete choice experiment, survey, tobacco, e-cigarettes, vaping, youth

## Abstract

**Objectives:** The novelty of e-cigarette regulatory policy poses difficulties for evidence-informed decision making because there is little evaluative evidence on the effects of specific policies. One way to provide evidence to inform Canadian policy in this situation is to learn from users how they would behave under different policy scenarios without actually implementing those policies in real-world settings. Discrete Choice Experiments provide an opportunity to undertake this research.

**Methods:** We recruited an online sample of 600 e-cigarette current and past users aged 16-25, using an existing panel of recently recruited e-cigarette users, to participate in a discrete choice experiment. Participants chose their preferred option from a choice of 2 e-cigarette products described by 4 attributes: flavour availability, location availability, nicotine concentration, and price.

**Results:** Our findings provide an overview of how important each attribute (price, nicotine concentration, availability, and flavour) is to young e-cigarette users. Across all features, as price increases, respondents were less willing to purchase. The study provides evidence that while all 4 attributes have strong effects, nicotine concentration and flavour most significantly influenced preferences for e-cigarettes.

**Conclusion:** This could provide points of comparison and a better understanding of how hypothetical regulatory restrictions could prevent youth uptake of e-cigarettes, encourage current youth vapers to quit vaping, and make e-cigarettes available and useful for smokers interested in vaping to help them completely quit combustible cigarette smoking.

## Introduction

With alarming uptake of e-cigarettes by youth and young adults and increasing calls to provide smokers with reduced risk products,^1^ Canada and other jurisdictions are grappling with difficult decisions about effective policy options to address this public health challenge. The novelty of e-cigarette regulatory policy poses difficulties for evidence-informed decision making because there is little evaluative evidence on the effects of specific policies.^2^ In reviewing the few studies of the effects of restrictions on flavours and of taxation, for instance, Siegel and Katchmar^3^ found the evidence to be inconclusive and context-specific. One way to provide evidence to inform Canadian policy in this situation is to learn from users how they would behave under different policy scenarios without actually implementing those policies in real-world settings. Discrete Choice Experiments provide an opportunity to undertake this research.

A discrete choice experiment (DCE) is a survey-based experimental approach with the objective of eliciting individual preferences for goods and services.^4^ In DCEs, the participants note a series of preferences across products described by a set of attributes and characteristics. The relative importance of each attribute and the value of alternative options can be derived from the choices, using choice models. This research strategy has significant precedent in both public health and tobacco research.^5-7^ As a hypothetical choice strategy, DCEs’ particular strengths are in their capacity to examine potential policies that have not yet been implemented. Past DCEs have found a range of important consumer preferences related to e-cigarettes, particularly concerning the conditions under which smokers prefer cigarettes and menthol cigarettes over e-cigarettes, as well as the impacts of price, flavour, and retail availability.^4,5,7,8^

Approximately 12% of Canadians are daily or occasional smokers,^9^ while approximately 6% of Canadians report e-cigarette use in the past 30 days.^10^ It is illegal to sell e-cigarettes to anyone in Canada under the age of 18, however some provinces have increased their age limits to 19 or 21.^11^ The Canadian government is presently considering a range of policies aimed at reducing both smoking and e-cigarette use. Health Canada recognizes the role of e-cigarettes in helping some individuals to quit smoking.^12^

To our knowledge, there has only been one Canadian consumer choice study focused on e- cigarettes, and this was performed in 2016.^13^ There have since been significant shifts in regulations and public knowledge in the intervening years. Discussions with key expert stakeholders from Ontario were conducted to further understand the priorities around regulations for e-cigarettes and youth uptake. From the literature review and stakeholder discussions, we identified the age group and the attributes to be emphasized.

## Methods

### Participants

We recruited an online sample of 600 recent and/or current e-cigarette users aged 16-25, using an existing panel of recently recruited e-cigarette users. Of the 600 participants recruited, 29 were excluded from the analytic sample for incomplete responses or failure to meet quality control criteria (eg, attention filter failures). This resulted in an analytic sample of N = 571. The age range was chosen because of the clear uptake of e-cigarettes at these ages.^14^ To be eligible, current users had to have used e-cigarettes on a regular basis for at least 6 months and be a Canadian resident between ages 16 and 25. Participants were recruited in March of 2021. Guardian consent was not required as it is largely accepted that youth may consent to minimal risk studies at 16 years of age.^15^

Inclusion criteria included ever users and current users of e-cigarettes, as well as ever dual users and current dual users of e-cigarettes and combustible cigarettes. Never smokers and never users were excluded from this study to avoid the risks of potentially increasing their chances of taking up e-cigarettes through exposure to information.

Sample size determination for DCE depends on a variety of factors including task complexity, sample composition, and the ideal statistical precision of findings.^16^ Notwithstanding, Hall et al^17^ have suggested that a good rule is 25 participants for each block of choices. Our 600 participants recruitment thus allowed for us to use a D-efficient survey design to allow for approximately 30 different choice sets.^18^ Beyond this, no power calculations were conducted. Respondents were randomised into 1 of 3 blocks of 10 choices, with each having different sets of scenarios viewed in different orders. The DCE itself was created through NGENE, a comprehensive software specifically designed for choice experimental designs. Price required a negative prior, so we used −.00001 to avoid dominant alternatives.

A survey was also administered to collect socioeconomic data, smoking, and e-cigarette use behaviour information on each respondent prior to the DCE. Our participant pool showed a more than 2:1 ratio of females to males – this is likely a result of those that were recruited through our original recruitment strategy. The majority (59.9%) of participants had completed high school. 94.9% (n = 542) of respondents had previously smoked cigarettes, with 31.2% (169) having smoked in the past 30 days. 82.4% had used an e-cigarette on 100 days or more, with the majority (55.4%) having vaped the past 7 days. 59.7% stated that they vaped all 7 days per week in a typical week. 96.9% of respondents had previously used flavours other than tobacco and 97.5% had used an e-cigarette with nicotine. A significant majority, 72.2% of participants, buy e-cigarettes from adult-only vape shops. The average cost of a single 30 mL bottle was $16.01, with the median at $15.00.

The survey was administered online and researcher contact information was provided if participants had any questions upon completion. Survey completion time was approximately 15 min. Each participant was compensated $10 for their time. All responses were anonymous and every participant completed an informed consent sheet.

### Design

The Federov algorithm created a candidate set (without dominant alternatives) consisting of 2000 feasible choice tasks (see Table A2).^19^ The algorithm then chose the 30 choice tasks that it deemed most efficient. The resulting design, including multinomial logit efficiency measures is presented in Appendix A. Our study has a theoretical foundation based on the random utility framework, which assumes maximum utility when respondents choose among 3 alternatives (E-cigarette A, E-cigarette B, or Neither).^20^ We used conditional logit models that require data to satisfy the Independence of Irrelevant Alternatives (IIA) assumption, which denoted that the presence of an additional alternative in the choice set should not alter the reference ranking.^21^ To empirically test the IIA assumption, we constructed conditional logit models using the full data and a subset of data excluding the ‘Neither’ option. Then we examined whether the IIA assumption held using the *Hausman-McFadden Test*, which provided test statistics that were used to summarize differences in the 2 models.^22^ This test compares the full model with a restricted model by excluding one alternative (eg, the ‘Neither’ option). Non-significant test statistics (*P* > .05) for all comparisons indicated that the IIA assumption was not violated, supporting the appropriateness of the conditional logit model for our data.

While the questionnaire used in this study was not a validated tool from previous literature, it was rigorously developed based on a comprehensive literature review and consultations with key stakeholders and experts. Additionally, we conducted a pilot test with 40 participants, representing approximately 7% of our final analytic sample (571 participants). The purpose of the pilot test was to evaluate the clarity, comprehensiveness, and functionality of the survey and discrete choice experiment. Feedback from the pilot participants was used to refine question phrasing and ensure the scenarios were easy to understand and aligned with the study objectives.

### Procedure methods

**Figure 1.**
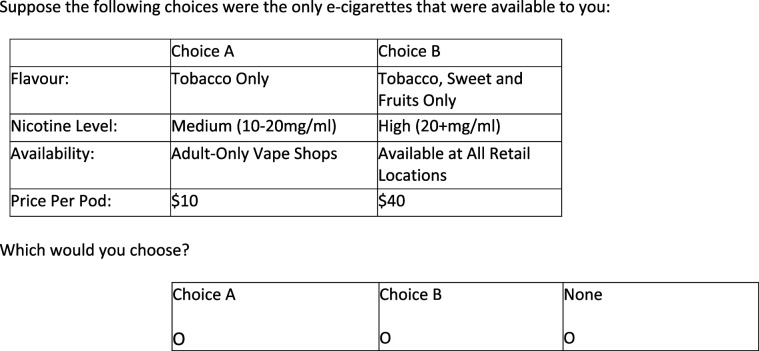
Example of a discrete choice scenario. For flavour, the reference group is “tobacco only”; for nicotine concentration, the reference group is “zero.”; for availability, the reference group is “prescription only”; and for price, the reference group is “$40.”

Respondents were asked to respond to scenarios by choosing their favourite option among e- cigarettes that each had 4 attributes^14^: flavour availability, location availability, nicotine concentration, and price (Table 1). Flavours were chosen to reflect currently available flavoured products in Canada: *Tobacco Only*; *Tobacco and Menthol Only*; *Tobacco*, *Sweet and Fruits Only*; *Tobacco*, *Menthol*, *Sweet and Fruits*. Availability is noted as *prescription-only*, *adult-only vape shops*, *online only*, and *available at all retail locations*. The levels of nicotine were High (20+ mg/ml), Medium (10-20 mg/mL), Low (1-9 mg/ml), and Zero. A level of ‘zero’ was provided, as nicotine-free options were available at retail. Finally, we defined price as the price paid for a pod or a 30 mL bottle. The price options ($10, $20, $30, and $40) were selected based on market research reflecting the typical range of prices for e-cigarette pods and 30 mL e-liquid bottles in Canada at the time of the study, with $40 representing a higher-than-average price to capture potential pricing scenarios. Each respondent would have 3 choices (Figure 1) – *E-cigarette A*, *E-cigarette B*, or *Neither*. The 4 attributes (flavor availability, location availability, nicotine concentration, and price) and their corresponding levels were pre-determined prior to the survey design based on a comprehensive review of the literature and input from expert stakeholders. These attributes were chosen because they are commonly examined in tobacco control research^4,23,24^ and are directly relevant to policy discussions surrounding e-cigarettes.

**Table 1.** DCE attributes and characteristics.

Attribute	Characteristics
Flavour availability	Tobacco only
	Tobacco and menthol only
	Tobacco, sweet and fruits only
	Tobacco, menthol, sweet and fruits
Availability	Prescription-only
	Adult-only vape shops
	Online only
	Available at all retail locations
Level of nicotine	High (20+ mg/ml)
	Medium (10-20 mg/mL)
	Low (1-9 mg/ml)
	Zero
Price (per pod or 30 mL bottle)	$10 CAD
	$20 CAD
	$30 CAD
	$40 CAD

Each participant was provided with 12 of these choice scenarios including 2 “dummy” questions (questions that simply asked participants to press a particular choice button in order to ensure participants were paying attention). Samples were split so that different sets went to different subgroups. In total, 3 choice sets (200 per set) were created. In order to increase the quality of the choice data collected, detailed narrative and visual information describing the e-cigarettes and their features were provided prior to the DCE. We used ‘forced responses’ to prevent respondents from skipping through the survey. Finally, we used attention filters embedded in the survey to check that respondents were paying attention (eg, identical questions – participants who answered differently for identical questions were excluded).

### Analysis

For our analysis, we used the following values as reference levels for attributes respectively (deemed to be the least attractive options): Flavour – Tobacco; Nicotine Concentration – Zero; Availability – Prescription-Only; Price – $40. Our analysis includes a conditional logit model using the 4 features only, and its corresponding willingness to pay (WTP) estimates (Table 2). WTP is the average marginal value that respondents are willing to pay for non-price attributes, given difference price conditions. In this analysis, average marginal effects refer to the estimated monetary value participants are willing to pay for non-price attributes, derived by dividing the coefficients of non-price attributes by the price coefficient. These effects represent the average trade-off participants make between price and other attributes across all choice sets in the study. We obtain precision estimates of WTP estimates using methods proposed by Krinsky and Robb.^25^ A *P*-value of .05 is used as the significance threshold for results interpretation.

**Table 2.** Willingness to pay (WTP) estimates for non-price features in main analysis.

Price	$30	$20	$10
Characteristic	WTP estimate [95% CI]	WTP estimate [95% CI]	WTP estimate [95% CI]
Flavour: tobacco, menthol	−1.04 [−1.51, −.73]	−.77 [−1.00, −.58]	−.62 [−.79, −.47]
Flavour: tobacco, menthol & fruit	−2.30 [−3.12, −1.78]	−1.71 [−2.08, −1.43]	−1.38 [−1.60, −1.19]
Flavour: tobacco, menthol, fruit and sweets	−2.46 [−3.29, −1.94]	−1.83 [−2.23, −1.53]	−1.47 [−1.69, −1.29]
Availability: online only	−1.25 [−1.74, −.92]	−.93 [−1.16, −.74]	−.75 [−.92, −.60]
Availability: adult-only vape shops	−1.71 [−2.30, −1.33]	−1.27 [−1.57, −1.04]	−1.02 [−1.19, −.87]
Availability: all retail locations	−1.74 [−2.41, −1.31]	−1.29 [−1.61, −1.05]	−1.04 [−1.24, −.87]
Nicotine: low	−1.63 [−2.25, −1.23]	−1.21 [−1.50, −.99]	−.97 [−1.16, −.82]
Nicotine: medium	−2.52 [−3.40, −1.97]	−1.87 [−2.29, −1.56]	−1.51 [−1.72, −1.32]
Nicotine: high	−2.56 [−3.42, −2.02]	−1.90 [−2.33, −1.59]	−1.53 [−1.75, −1.34]

Note. The reference groups are as follows: flavour – tobacco; nicotine concentration – zero; availability – prescription-only; price – $40.

Note: CI, confidence interval.

To incorporate potential impacts of SES correlates, we conducted sensitivity analysis by building a conditional logit model with features and their interactions with SES correlates including gender, education, last time the respondents vaped, having ever used flavoured e-liquid or pods, and having vaped in the last 30 days. To compare models with features only and their counterparts with interactions, we use likelihood ratio tests and Akaike Information Criterion (AIC). The decision threshold (*P*-value) for likelihood ratio test is .2. The model with lower AIC values is considered as the preferrable model. IIA testing suggested passing of the IIA assumption test.^26^ Both AIC and likelihood ratio test indicated that non-interaction model was preferred, we are therefore presenting the model with attributes only. We have, however, included the interaction models in an appendix.

## Results

*Conditional logit model with features only*: Our analysis includes a conditional logit model using the 4 features only, and its corresponding willingness to pay (WTP) estimates (Table 2). Conditional logit model results show that participants derive positive utility from all 4 features and all 12 characteristics (all *P-values* <.001). The attributes are as follows: flavour - tobacco, menthol, fruit and sweets; availability - being available at all retail locations; nicotine level - high; and price - $10. The odds ratio indicates the increased odds that participants would choose that characteristic in comparison to the constant. For instance, the odds of participants choosing a flavour of “Tobacco, Menthol” were 1.8 times higher than the odds of choosing “Tobacco,” while participants’ odds of choosing “Tobacco, Menthol, Fruit, and Sweets” were 4.01 times higher than “Tobacco.”

The odds ratios for the attributes and their levels are summarized in Table 4. Across all features, as price increases, respondents are less willing to purchase; they demonstrate significant and negative WTPs for all the non-price features, regardless of price (see Table A1). Respondents willing to purchase at lower prices only at $10 or $30 (ie, have positive WTPs) with the following attributes: tobacco, menthol flavor; being available online only; low nicotine level. When the price is $20, respondents show negative WTPs to the above-mentioned attributes, compared to a price of $40. All the estimates are significant except for those being available at adult-only-vape shops or all retail locations across price of $10, $20, and $30, as well as at “Low nicotine level” where price is $30.

*Conditional logit model with features and their interactions with SES correlates*: Demographic characteristics of the sample are summarized in Table 3. As we introduce interactions between features and SES correlates, choice probabilities are only significantly affected by: tobacco, menthol, fruit flavor; tobacco, menthol, fruit and sweets flavor; and medium levels of nicotine (Table 2, *P*-values <.05). There were multiple interactions found to be significant. When the nicotine level is high or price was $10, different education levels affected choice probabilities. Both medium and high nicotine levels interacted with gender, last time respondents vaped, and having ever used flavoured e-liquid or pods. Differences in last time respondents vaped also vary choice probabilities attributable to price of $30, $20, and $10. For gender, males demonstrated significantly higher odds of choosing flavors with broader appeal, such as “Tobacco, Menthol, Fruit and Sweets” (OR: 1.37, *P* < .001) compared to females. Similarly, males were more likely to select medium and high nicotine concentrations (OR: .71 and .74, *P* < .001, respectively), indicating a stronger preference for higher nicotine levels. For education, participants with higher education levels were less likely to prefer high nicotine concentrations (OR: .87, *P* = .004) but showed no significant differences in preferences for flavors or availability attributes. This suggests that educational background may influence nicotine-related choices rather than flavor or retail availability.

**Table 3.** Demographic characteristics.

Characteristic	Overall (N = 571)
Age	
16-18	154 (27%)
19-21	233 (40.8%)
22-24	184 (32.2%)
Education	
Some high school	82 (14.4%)
High school	342 (59.9%)
Bachelor’s degree	109 (19.1%)
Master’s degree	2 (.4%)
Trade school	29 (5.1%)
Prefer not to say	7 (1.2%)
Gender	
Male	148 (25.9%)
Female	368 (64.4%)
Non-binary	46 (8.1%)
Prefer not to say	3 (.5%)
Prefer to self-describe	6 (1.1%)
Last time vaped	
Earlier today	242 (42.4%)
Not today but sometime in the past 7 days	74 (13.0%)
Not in the past 7 days but sometime in the past 30 days	54 (9.5%)
Not in the past 30 days but sometime in the past 6 months	101 (17.7%)
Not in the past 6 months but sometime in the past year	41 (7.2%)
1 to 4 years ago	55 (9.6%)
5 or more years ago	4 (.7%)
Have ever used flavoured e-liquid or pods	
Yes	555 (97.2%)
No	16 (2.8%)
Have you used an e-cigarette or vaped in the past 30 days?	
Yes	376 (65.8%)
No	195 (34.2%)
Where do you buy the e-cigarette/vaping device, pods or e-liquid from?	
Adult-only vape shop	319 (72.2%)
Convenience store	188 (42.5%)
Gas station	159 (36.0%)
Online	109 (24.7%)
Prefer not to answer	9 (2.0%)
Other	26 (5.9%)
Have you ever smoked cigarettes?	
Yes	542 (94.9%)
No	29 (5.1%)
Have you smoked cigarettes in the past 30 days?	
Yes	169 (31.2%)
No	373 (68.8%)

How much did you pay for a 30 mL e-liquid bottle or single pod?												
Total count (N)	Min	Max	Mean	StDev	Sum	Percentile						
						.05	.10	.25	.50 median	.75	.90	.95
380	3.00	40.00	16.01	7.20	6085.27	5.00	5.00	10.00	15.00	20.00	25.00	25.20

While the estimated odds ratios in Table 4 suggest certain features, such as flavor profiles and nicotine concentration, may have stronger effects, overlapping 95% confidence intervals indicate no statistically significant difference between some characteristics. For example, no significant difference was observed between ‘menthol and fruit’ and ‘menthol, fruit, and sweets,’ between ‘adult-only vape shops’ and ‘all locations,’ or between ‘medium’ and ‘high’ nicotine levels. As such, interpretations regarding the most important features should be made with caution and based on the magnitude of the estimated ORs, rather than statistical significance alone.

**Table 4.** Determinants of e-cigarette choices.

Characteristic	OR	95% CI	*P*-value
Constant flavour: Tobacco			
Flavour: Tobacco, menthol	1.80	1.58, 2.05	<.001
Flavour: Tobacco, menthol & fruit	3.66	3.21, 4.17	<.001
Flavour: Tobacco, menthol, fruit and sweets	4.01	3.53, 4.56	<.001
Constant availability: Prescription only			
Availability: Online only	2.03	1.79, 2.29	<.001
Availability: Adult-only vape shops	2.62	2.32, 2.96	<.001
Availability: All retail locations	2.66	2.36, 3.01	<.001
Constant nicotine: Zero			
Nicotine: Low	2.50	2.20, 2.85	<.001
Nicotine: Medium	4.14	3.63, 4.72	<.001
Nicotine: High	4.23	3.70, 4.83	<.001
Constant price: $40			
Price: $30	1.76	1.53, 2.02	<.001
Price: $20	2.14	1.87, 2.45	<.001
Price: $10	2.57	2.32, 2.84	<.001

Note. OR, odds ratio; CI, confidence interval.

For flavour, the reference group is “tobacco only”; for nicotine concentration, the reference group is “zero.”; for availability, the reference group is “prescription only.”; and for price, the reference group is “$40.”

## Interpretation

Depending on regulators’ objectives, these data can be interpreted in a range of ways. If the goal of regulators is to decrease youth use of e-cigarettes, flavour should be restricted to tobacco only, nicotine concentrations to low (or none), availability to prescription only, and price to $40. Medium and high nicotine concentrations (OR 4.14 and 4.23) are particularly important for young vapers, followed closely by availability of flavours that include fruit and sweets alongside menthol and tobacco (OR 4.01). Still highly important are price and availability. Although price and availability have lower odds (ranging from 1.76 to 2.66), the various levels of these 2 characteristics are significantly higher than their respective constants. Moreover, some ORs within a given characteristic are of note: Medium Nicotine Concentration (10-20 mg) was only .09 less likely to be chosen than High Nicotine; while Tobacco, Menthol, and Fruit was only .35 more likely to be chosen than Tobacco, Menthol, Fruit and Sweets. The more significant jumps were between Medium vs Low Nicotine Concentrations (1.64) and between Tobacco, Menthol, and Fruit vs Tobacco, Menthol (1.86).

Our study contributes to the growing body of evidence examining e-cigarette preferences, particularly among youth and young adults, and highlights several findings that align with and expand upon previous discrete choice experiments (DCEs) conducted with adult smokers. Similar to the work by Shang et al^7^ and Marti et al,^27^ we find that non-price attributes such as flavor and nicotine concentration play a significant role in shaping preferences, particularly for youth, who are drawn to sweet and fruity flavors as well as higher nicotine concentrations . However, unlike studies focusing on adult smokers, such as Pesko et al^28^ and Buckell et al,^4^ where health benefits and cessation potential were key drivers of e-cigarette choice, our findings suggest that for youth, the appeal of flavors and nicotine strength outweighs considerations of harm reduction or smoking cessation.

The importance of flavor preferences has been a consistent finding across discrete choice experiment studies. For instance, Shang et al^24^ found that menthol flavors were less appealing to adult smokers, while our study highlights the opposite for youth, where sweeter flavor profiles including menthol were highly valued. These findings underscore the need for differentiated regulatory approaches that account for how flavor preferences vary by age and smoking status.

Nicotine concentration preferences also reveal a divergence between youth and adult populations. Similar to our study, Marti et al^27^ observed that higher nicotine levels are particularly valued by those who both smoke and use e-cigarettes. This aligns with our observation that the high prevalence of dual use (approximately one-third of our sample) possibly amplified preferences for high nicotine concentrations.

Our findings also align with the broader literature in demonstrating a negative relationship between price and likelihood of purchase.^27,28^ Yet the strength of this relationship was not nearly as strong. For instance, while Shang et al^24^ found significant willingness to pay (WTP) for less harmful attributes among adult smokers, our study found that price played a relatively modest role in youth preferences compared to flavors and nicotine strength. This suggests that price-focused policies, such as taxes on e-cigarettes, may have less impact on reducing youth uptake than flavor or nicotine regulations.

Overall, our findings contribute to the broader evidence base by demonstrating that while many preferences overlap between youth and adults, the motivations underlying these preferences differ significantly. Policymakers should consider these differences to craft tailored regulations that protect youth while supporting harm reduction for adult smokers.

Given these findings, policymakers aiming to reduce youth e-cigarette use could focus on regulating the factors with the highest odds ratios, such as nicotine concentration and flavor availability, as these appear to have the greatest influence on young users’ choices. Additionally, while this analysis does not cover the potential impacts on adult smokers using e-cigarettes for cessation, any regulatory changes should consider these dual objectives to avoid unintended consequences that might hinder smoking cessation efforts. Finally, it will be important to consider the implications of any regulations on underground e-cigarette markets in order to avoid regulations that simply push e-cigarette users to unregulated and less safe options.

## Limitations

First, data collected may be prone to hypothetical bias,^29^ meaning the preferences indicated in survey may deviate from real-life behavior.

Second, while this provides information on what individuals “prefer,” it does not provide insights on what they would do if that product was not available. It remains possible that respondents would quit nicotine entirely, switch to the illicit market, or even switch over to combustible cigarettes. It was not within the confines of our research to evaluate how users would respond to such restrictions, but such differences in behaviour would have vastly different policy implications from a public health perspective.

Third, the high prevalence of dual use and past smoking among participants likely influenced their preferences for e-cigarette attributes. Dual users may be more inclined toward higher nicotine concentrations or certain flavors that align with their existing smoking behaviors, which could partially explain the strong preference for nicotine and flavors observed in our study.

Finally, our research did not evaluate how these options would impact individuals who smoke, individuals who both smoke and vape, and individuals who only vape. Such differences are important in terms of policymakers’ goals in reaching particular populations, while not pushing others to illicit markets.

## Conclusion

Our findings provide an overview of how important each attribute (price, nicotine concentration, availability, and flavour) is to young e-cigarette users. In the absence of rigorous real-world evidence on the effects of e-cigarette policies, the results are a good proxy for the likely effects of current and new policy alternatives being contemplated. The study provides evidence that while all 4 attributes have strong effects, nicotine concentration and flavour most significantly influenced preferences for e-cigarettes. Further research should be done on understanding non-cigarette and cigarette users’ attribute preferences. Future research could provide points of comparison and a better understanding of how regulatory policy change could prevent youth uptake of e-cigarettes, encourage current youth vapers to quit vaping and make e-cigarettes available and useful for smokers interested in vaping to help them completely quit combustible cigarette smoking.

## Appendix

### Appendix A

**Table A1.** Willingness to pay (WTP) Estimates for Non-price Features.

Price	$30	$20	$10
Characteristic	WTP estimate [95% CI]	WTP estimate [95% CI]	WTP estimate [95% CI]
Flavour: Tobacco, menthol	1.41 [−12.74, 13.96]	1.14 [−9.19, 10.75]	6.28 [−25.56, 31.01]
Flavour: Tobacco, menthol & fruit	2.07 [−20.07, 19.31]	1.68 [−13.26, 14.96]	9.23 [−36.88, 42.07]
Flavour: Tobacco, menthol, fruit and sweets	2.26 [−23.23, 22.01]	1.83 [−14.03, 15.55]	10.07 [−38.12, 45.01]
Availability: online only	1.60 [−14.85, 14.36]	1.30 [−10.28, 13.29]	7.11 [−29.70, 45.01]
Availability: Adult-only vape shops	1.37 [−13.16, 14.01]	1.11 [−9.27, 11.37]	6.08 [−28.43, 26.19]
Availability: All retail locations	1.79 [−14.97, 15.12]	1.45 [−11.59, 13.43]	7.96 [−35.55, 33.22]
Nicotine: Low	1.19 [−11.78, 11.09]	.97 [−8.63, 10.59]	5.31 [−23.74, 24.09]
Nicotine: Medium	2.63 [−26.37, 25.09]	2.13 [−18.74, 21.17]	11.71 [−48.06, 53.48]
Nicotine: High	1.91 [−19.08, 18.58]	1.55 [−12.21, 16.08]	8.51 [−35.83, 36.79]

Note. The reference groups for the conditional logit model are as follows - flavor: tobacco only; nicotine concentration: zero nicotine; availability: prescription only; price: $40. For interactions with SES variables, the reference groups are: gender: female; education: high school or less; last time vaped: more than 30 days ago. Odds ratios represent the relative likelihood of selecting an attribute or combination of attributes compared to these baseline categories.

Note. CI, confidence interval.

**Table A2.** DCE candidate set.

MNL efficiency measures										
D error	0.210921									
A error	0.37118									
B estimate	99.99976									
S estimate	18872381									
Prior	Flavour (d0)	Flavour (d1)	Flavour (d2)	Availability (d0)	Availability (d1)	Availability (d2)	Nicotine (d0)	Nicotine (d1)	Nicotine (d2)	Price
Fixed prior value	.001	.002	.003	.001	0	.002	.001	.002	.003	−.00001
Sp estimates	1564809	395141.1	197113.8	1573389	Undefined	401087.8	1599909	383337.3	172448.1	18872381
Sp t-ratios	.001567	.003132	.004476	.001563	0	.003095	.00155	.003166	.00472	.000143
Choice situation	ecig1.flavour	ecig1.availability	ecig1.nicotine	ecig1.price	ecig2.flavour	ecig2.availability	ecig2.nicotine	ecig2.price	Block	
1	0	2	3	3	3	1	3	10	1	
2	2	3	2	7	3	0	0	5	1	
3	3	1	3	3	0	1	2	7	2	
4	3	3	2	3	3	1	2	3	2	
5	0	2	0	5	3	3	2	10	3	
6	3	1	2	10	2	1	3	3	3	
7	2	3	0	7	3	1	0	10	1	
8	0	3	1	3	3	1	3	5	1	
9	1	3	0	7	3	2	1	10	1	
10	3	0	2	5	3	2	3	3	1	
11	2	3	1	3	1	2	0	7	2	
12	3	2	3	3	0	1	2	10	2	
13	1	0	2	7	3	3	0	3	2	
14	3	3	1	3	2	0	3	5	3	
15	0	2	3	10	3	1	2	10	3	
16	3	1	2	3	1	2	3	3	1	
17	2	3	2	10	3	1	3	5	1	

18	0	3	3	3	1	2	0	7	1	
19	3	2	2	3	3	3	1	10	2	
20	1	3	3	7	2	0	2	3	2	
21	3	3	0	3	1	2	1	5	2	
22	2	1	3	3	3	3	2	10	3	
23	3	3	1	3	1	2	0	7	3	
24	0	2	3	10	3	3	2	10	1	
25	3	1	3	3	1	2	0	7	1	
26	1	3	2	3	3	3	1	5	1	
27	3	2	3	10	2	0	2	3	2	
28	2	3	0	3	3	1	2	7	2	
29	1	0	3	3	2	3	2	10	2	
30	0	1	3	5	3	3	0	10	2	
